# Clinical characteristics and programmed cell death ligand-1 expression in adenocarcinoma *in situ* and minimally invasive adenocarcinoma of lung

**DOI:** 10.18632/oncotarget.22082

**Published:** 2017-10-26

**Authors:** Renke Yu, Zhengfu He, Ying Lou, Hanliang Jiang, Yuhui Wu, Zhen Liu, Hongming Pan, Weidong Han

**Affiliations:** ^1^ Department of Medical Oncology, Sir Run Run Shaw Hospital, College of Medicine, Zhejiang University, Hangzhou, Zhejiang, China; ^2^ Department of Thoracic Surgery, Sir Run Run Shaw Hospital, College of Medicine, Zhejiang University, Hangzhou, Zhejiang, China; ^3^ Department of Respiratory Medicine, Sir Run Run Shaw Hospital, College of Medicine, Zhejiang University, Hangzhou, Zhejiang, China

**Keywords:** adenocarcinoma in situ, minimally invasive adenocarcinoma, programmed cell death ligand-1, clinical characteristics, lung adenocarcinoma

## Abstract

**Objectives:**

According to the IASLC/ATS/ERS 2011 classification, there are two new conceptions of lung adenocarcinoma, adenocarcinoma *in situ* (AIS), minimally invasive adenocarcinoma (MIA), which are very early stages of lung adenocarcinoma. This study aimed to analyze clinical features of AIS and MIA and determine the expression profile of PD-L1 in AIS and MIA.

**Results:**

In all 274 patients, 77 were diagnosed as AIS and 197 as MIA. We accidentally found 4 patients with recurrence, which were all MIA. The median age of the patients at diagnosis was both 52 years. 71.4% were female in AIS as while as 71.1% in MIA. 36.4% patients were observed with ever symptoms in AIS and 28.9% in MIA. 12.9% and 8.6% had smoking history respectively in AIS an MIA. All AIS and MIA cases were PD-L1 negative. There was significant association between symptoms and more mild progression of nodules in chest CT before surgery.

**Materials and Methods:**

We analyzed some clinical features of 274 patients including age, sex, smoking history, family history, surgery, EGFR mutation, ALK, ROS-1, serum CEA level *et al*. The expression of PD-L1 was evaluated by immunohistochemical analysis in 37 specimens of MIA and 17 specimens of AIS.

**Conclusions:**

There are no significant differences between AIS and MIA in clinical features. AIS and MIA almost do not express PD-L1 protein and without any lymph node metastasis. The surgery intervention is supposed to be as small as possible.

## INTRODUCTION

Lung cancer is the second leading cancer type for the estimated new cancer cases and the leading cause of cancer death in the United States in 2016 [1]. The high incidence and mortality of lung cancer are always catching the attention of scientists, and naturally the research about lung cancer becomes deeper and more elaborate. According to the International Association for the Study of Lung Cancer/American Thoracic Society/European Respiratory Society international multidisciplinary classification 2011 (IASLA/ATS/ERS 2011) of lung adenocarcinoma, adenocarcinoma *in situ* (AIS) is defined as a tumor that grows in a lepidic fashion along pre-existing airway structures without detectable invasion. Minimally invasive adenocarcinoma (MIA) is defined as adenocarcinoma ≤3 cm with a predominantly lepidic pattern and ≤5 mm invasion in the greatest dimension [2]. According to the National Comprehensive Cancer Network (NCCN) Guidelines Version 4.2017 Non-Small Cell Lung Cancer, for AIS or MIA patients, we usually give surgical exploration and resection plus mediastinal lymph node dissection or systematic lymph node sampling. More and more incidentally discovered pulmonary nodules were found though low-dose computed tomography (CT), most of which were early stage of lung cancer, like AIS or MIA [3]. Several studies have revealed some molecular features of AIS/MIA. The frequency of Epidermal Growth Factor Receptor (EGFR) mutation was AIS (62%), and MIA (60%) [4]. And Sato *et al* reported the EGFR mutation rate of AIS with smoking was 61% [5]. But the researches specific for AIS/MIA are still not enough, especially for patients in China, and the number of AIS/MIA cases studied in existing reports is small. One of the intractable problem for AIS/MIA is the indication for surgery and how long to follow up before surgery. There is absolute need to do some research about AIS/MIA of Chinese patients.

Programmed cell death-1 (PD-1) is an immunosuppressive receptor of the CD28/CTLA-4 family, which negatively regulates antigen receptor signaling by recruiting protein tyrosine phosphatase upon interacting with its ligand (PD-L1) [6]. Tumor cells hide some antigen features though the interaction between PD-1 and PD-L1, resulting in something like immune escape. Accumulating evidences demonstrated that PD-L1 overexpression is associated with poor prognosis in many kinds of malignant tumor, including lung cancer [6]. Scientists have paid huge attention on PD-L1 and several clinical trials have been carried out, some of which revealed that anti-PD-L1 therapy even could replace chemotherapy in lung cancer [7–12]. For example, Nivolumab can prolong overall survival (OS) in the patients with ever treated, squamous-cell lung cancer, compared with docetaxel chemotherapy, no matter PD-L1 positive or not [8]. But Nivolumab couldn't prolong the disease-free survival (DFS) in patients with non-squamous-cell lung cancer [7]. Another anti-PD-L1 antibody came out. Pembrolizumab prolongs OS and has a favourable benefit-to-risk profile in patients with previously treated, PD-L1-positive, advanced non-small-cell lung cancer [10]. However, the situation of the expression of PD-L1 in the AIS/MIA hasn't been reported clearly and finding some relationship between PD-L1 expression and low-invasion of AIS/MIA is in a great need.

In this study, we analyzed the clinical characteristics and the expression of PD-L1 in 274 AIS or MIA patients from the Sir Run Run Shaw (SRRS) Hospital, College of Medicine, Zhejiang University, China.

## RESULTS

### Clinical features

The clinical characteristics of 274 patients are shown in Table 1. In these patients, 77 were diagnosed as AIS while 197 were MIA according to the IASLC/ATS/ERS 2011 classification. The detailed information about the 274 patients was listed in Supplementary Table 1. 4 MIA patients were accidentally found with recurrence and underwent the second pulmonary resection and all found to be invasive adenocarcinoma at the recurrence. The median age of the patients at diagnosis of AIS or MIA was both 52 years, ranging from 25 to 80, 27 to 82 respectively. 55 (71.4%) were female in AIS as while as 140 (71.1%) in MIA, which were almost the same. 28 (36.4%) patients were observed with ever symptoms in AIS and 57 (28.9%) in MIA, like coughing, expectoration, chest tightness, chest pain, blood-stained sputum and so on. As for smoking, 10 (12.9%) and 17 (8.6%) had smoking history respectively in AIS and MIA. 19 (24.7%) of AIS were found to have related past history or family history, which was 56 (28.9%) in MIA.

**Table 1:** The clinical characteristics of AIS/MIA

	AIS	MIA	*P* value
Sum	77	197	
Age (range, years)	(25–80)	(27–82)	0.795
>52	39	98	
<52	38	89	
Sex			
Men	22	57	0.745
Female	55 (71.4%)	140 (71.1%)	
Smoking			
ever	10	17	0.277
never	67	180	
Symptom			
ever	28	57	0.232
none	49	140	
Past history or family history			
ever	19	56	0.531
none	58	141	

### Surgery information

**Figure 1:**
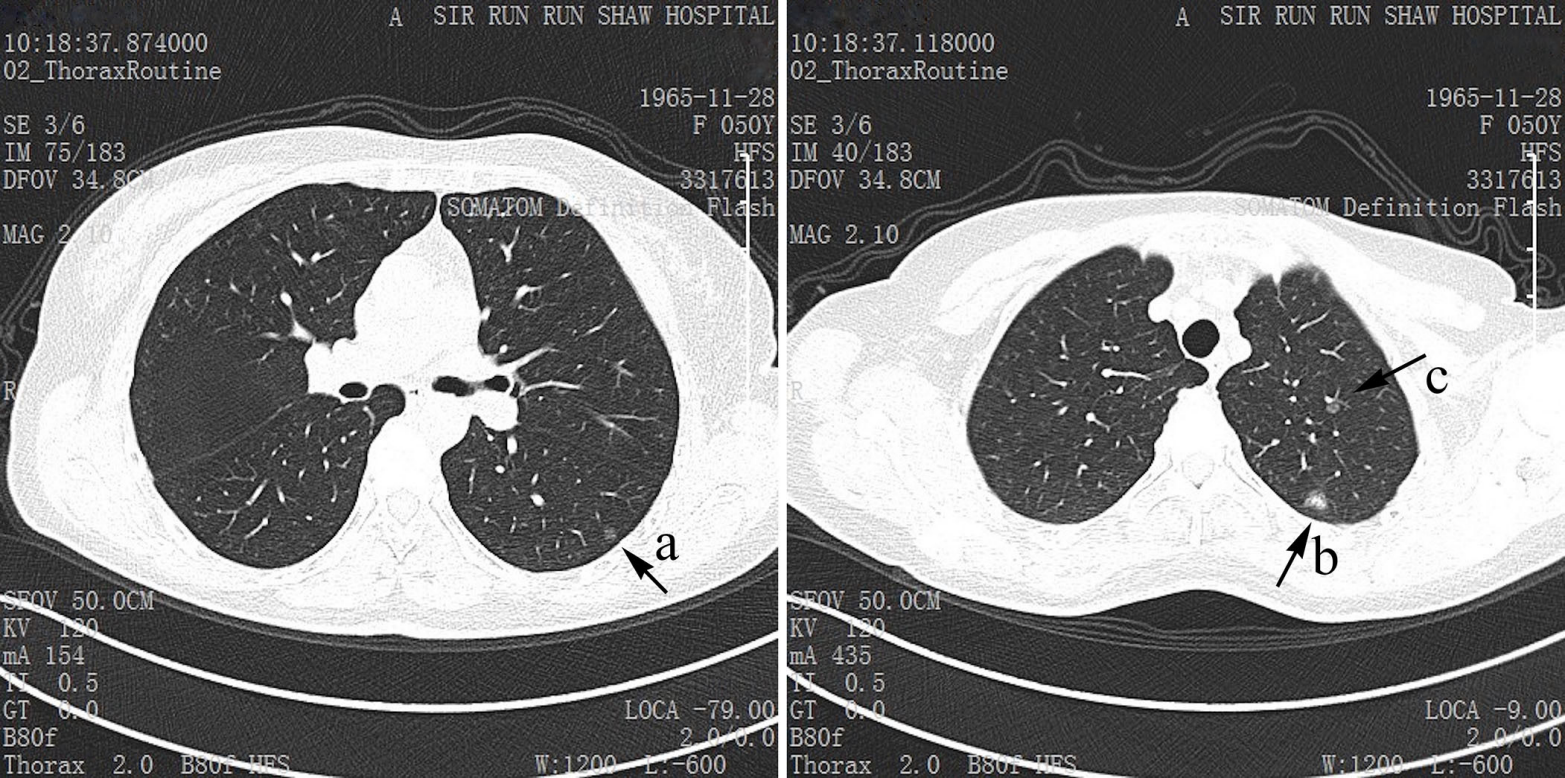
The typical CT images of AIS and MIA The nodule a and c are typical AIS, and the larger nodule b in the left upper lobe is MIA.

The surgery information is shown in Table 2. In cases of AIS, 70.1% underwent wedge resection, 14.3% segmentectomy, 15.6% lobectomy; while in cases of MIA, 67.0% underwent wedge resection, 23.4% segmentectomy, 9.6% lobectomy. There was difference in the distribution of location. In AIS, 30.3% nodules were located in right upper lobe (RUL), 9.0% in right middle lobe (RML), 13.5% in right lower lobe (RLL), 30.3% in left upper lobe (LUL), and 16.9% in left lower lobe (LLL). In MIA, 36.5%, 8.3%, 17.8%, 23.7%, 13.7% were respectively located in each lobe. We observed the tumor size of 35 cases (45.5%) of AIS and 103 cases (52.3%) of MIA larger than 0.8 cm. 8 (10.4%) of AIS were multiple nodules while 35 (17.8%) of MIA. Lymph node sampling was done in 38 AIS cases and 115 MIA cases, ranging from one node to 61 lymph nodes, resulting in none lymph node metastasis. The typical images of AIS or MIA in the chest CT are shown in Figure 1.

**Table 2:** The surgery information

	AIS	MIA	*P* value
Surgery			
wedge resection	54 (70.1%)	132 (67.0%)	NA
segmentectomy	11 (14.3%)	46 (23.4%)	
lobectomy	12 (15.6%)	19 (9.6%)	
Location			
RUL	27 (30.3%)	88 (36.5%)	NA
RML	8 (9.0%)	20 (8.3%)	
RLL	12 (13.5%)	43 (17.8%)	
LUL	27 (30.3%)	57 (23.7%)	
LLL	15 (16.9%)	33 (13.7%)	
Tumor size			
≥0.8 cm	35	103	0.268
<0.8 cm	42	94	
Multiple nodules			
Yes	8 (10.4%)	35 (17.8%)	0.131
No	69	162	
Lymph node sampling			
metastasis	0	0	NA
Non-metastasis	38	115	
No sampling	39	82	

RUL, right upper lobe; RML, right middle lobe; RLL, right lower lobe; LUL, left upper lobe; LLL, left lower lobe; NA, not applicable.

### Laboratory examination

**Figure 2:**
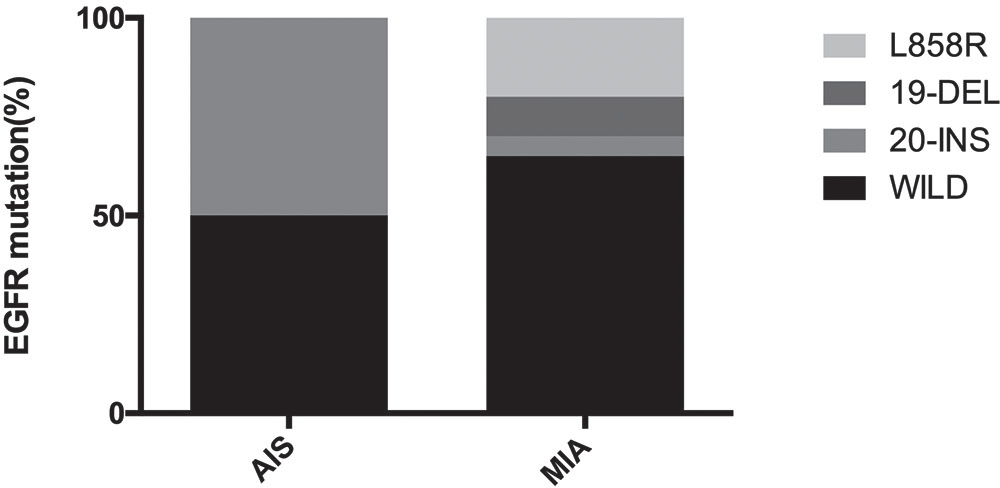
The EGFR mutation status in AIS and MIA In AIS, 50% were wild type and 50% were 20-Insert mutation. In MIA, 20% were L858R mutation, 10% were 19-Deletion, and 5% were 20-Insert mutation, 65% were wild-type.

The laboratory examination is shown in Table 3. We found 2 patients of AIS detected with EGFR gene and only 1 with 20-Insert mutation, and 20 patients of MIA detected with EGFR gene and 4 (20%) with L858R mutation, 2 (10%) with 19-Deletion, and 1 (5%) with 20-Insert mutation, leaving 13 (65%) wild-type. The result of EGFR mutation is shown in Figure 2. There were 23 patients (2 AIS, 21 MIA) that underwent the IHC analysis of anaplastic lymphoma kinase (ALK) with the result of all negative. There were 17 patients (1 AIS, 16 MIA) who underwent IHC analysis of ROS-1 with the result of 1 AIS negative, 9 MIA negative and 7 MIA positive. None of these patients were found abnormal serum carcinoembryonic antigen (CEA)level at the time of diagnosis and only 5 patients presented higher CEA during the follow-up. No significant association between six factors (sex, age, symptoms, past or family history, smoking and tumor size) with EGFR mutation was observed, which is shown in Table 4.

**Table 3:** The laboratory examination of AIS/MIA

	AIS	MIA	*P* value
EGFR mutation			
L858R mutation	0	4 (20%)	
19-Del	0	2 (10%)	
20-Ins	1	1 (5%)	
Sum of mutatuion	1 (50%)	7 (35%)	0.674
Wild	1	13 (65%)	
IHC			
ALK(–)	2	21	
ALK(+)	0	0	
ROS-1(–)	1	9	0.388
ROS-1(+)	0	7	
Serum CEA level			
Average (ug/L)	1.7	1.53	
Normal	54	138	0.164
Abnormal	0	0 (5 during follow-up)	

**Table 4:** The univariate analysis of EGFR mutation

	EGFR mutation	Wild type	*P* value
Sex			
Male	2	3	1.000
Female	7	9	
Age			
≥52 years	5	8	0.673
<52 years	4	4	
Symptoms			
ever	3	2	0.611
never	6	10	
Past or family history			
ever	2	5	0.642
never	7	7	
Smoking			
ever	0	1	1.000
never	9	11	
Tumor size			
≥0.8 cm	6	5	0.387
<0.8 cm	3	7	

### PD-L1 expression

**Figure 3:**
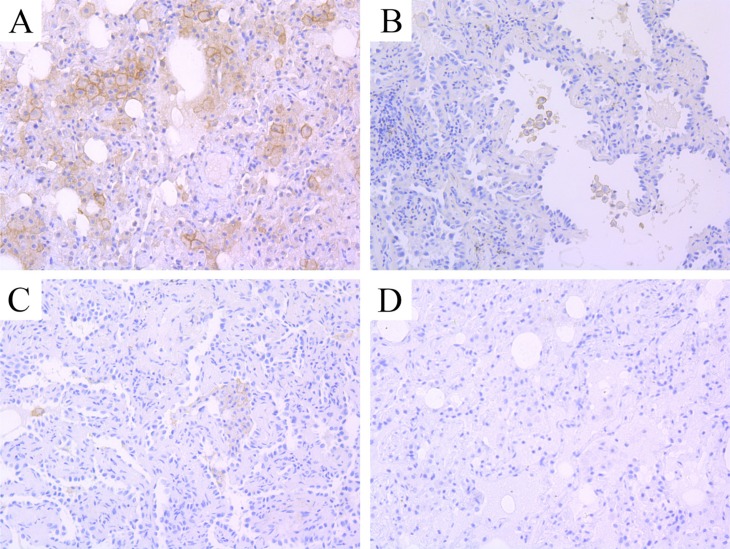
The typical IHC images of PD-L1 expression (**A**) positive staining; (**B**–**D**) negative staining of PD-L1.

All AIS and MIA cases were PD-L1 negative. The typical IHC of positive PD-L1 expression was like Figure 3A, which was gotten from the Pathology department of SRRS Hospital. And negative expression of PD-L1 was Figure 3B–3D.

### Follow-up data

The follow-up data is shown in Table 5. The follow-up chest CT scans before and after surgery were retrospectively analyzed. There were follow-up data of 30 AIS and 51 MIA before surgery, finding the mean follow-up time was 9.8 months of AIS and 18.1 months of MIA before surgery. In cases of AIS, 5 cases were observed with mild progression in the chest CT scan, which meant nodules became a little bit larger, or more obvious, or the density increased, while 14 cases in MIA. After surgery, 62 AIS (mean follow-up time 15.8 months) and 168 MIA (mean follow-up time 18.9 months) were followed up, finding 9 AIS and 22 MIA with mild progression in the CT scan, which meant new nodules came out or residual nodules became larger.

**Table 5:** The follow-up data before and after surgery of AIS/MIA

	AIS	MIA	*P* value
Before surgery			
Sum/mean time	30/9.8 months	51/18.1 months	
Progression^*^	5 (16.7%)	14 (27.5%)	0.269
Non-progression	25	37	
After surgery			
Sum/mean time	62/15.8 months	168/18.9 months	
Recurrence	0	4	
Progression^**^	9 (14.5%)	26 (15.4%)	0.857
Non-progression	53	142	

^*^Nodules became a little bit larger, or more obvious, or the density increased.

^**^New nodules came out or residual nodules became larger.

### Univariate analysis results for progression

Eight clinical and pathological variables were subjected to univariable analysis, including age, sex, tumor size, multiple nodules, smoking history, past history or family history, symptoms and pathology type. The result is shown in Table 6. We found significant association between symptoms and more mild progression of nodules in the chest CT during the follow-up before surgery.

**Table 6:** Univariate and multivariate analysis results for progression

Variable	Univariate Analysis	
	chi-square value	*P* value
Age, (≥52 VS <52 years)	1.368	0.242
Sex, (female VS male)	0.273	0.601
Tumor size, (≥0.8 VS <0.8 cm)	1.011	0.315
Multiple, (yes VS no)	0.272	0.602
Smoking, (yes VS no)	1.739	0.187
Past history, (yes VS no)	0.120	0.914
Symptoms, (yes VS no)	5.039	0.025^*^
Pathology type, (MIA VS AIS)	1.224	0.269

## DISCUSSION

In a systematic analysis of AIS/MIA reported by Madhusmita Behera (M.B.) in 2016 [13], the median age of patients at diagnosis of AIS/MIA estimated was 65.5 years and 64% of these patients were female and 40% were smokers. In this study, the median age of patients at diagnosis of AIS/MIA was 52 years, which is much earlier than former reported. The studies analyzed by M.B. were all published before 2014, meaning the patients included in these studies got the diagnosis of AIS/MIA before 2014, which is earlier than our study. As the quick development of the technology of CT and the wide application of low-dose CT in the lung screen and people's bigger concern about health, nodules in the lung can be found at a relatively younger age, which was proofed by the American National Lung Screening Trial Research Team [3]. But we can't deny the age of onset is being earlier due to some environment and lifestyle factors.

The female patients in this study were much more than male, and the ratio of smoking patients was significantly less than that of lung cancer. This result was consistent with M.B.'s report, but with higher female ratio and less smoking ratio. It may be partially due to the factor of sex, but we can't ignore the mental factors that women are easier to get worried or anxious about health as well as to accept chest CT exam or surgery. There were only 12.9% of AIS patients and 8.6% MIA patients with smoking history. Paige M. Bracci *et al* [14] did a large case-control study and found there was an association between AIS and smoking that was smaller in magnitude than other subtypes of non-small cell lung cancer. In this study, there was no significant association between smoking and more mild progression of nodules in chest CT before surgery. More researches should be carried out to demonstrate the relationship between smoking and AIS/MIA.

According to M.B.'s report, the 5-year DFS rate of AIS and MIA was respectively 100% and 96.7%, and the 5-year OS was respectively 100% and 98.5%. There was no significant difference of DFS or OS between these two tumor types [13]. In this study, only 4 cases of MIA with recurrence and no AIS were found with recurrence, which is consistent with former reports. But cause many cases in our study are so fresh that haven't reach 5 years or outcome event, no DFS or OS rate can be figured out yet.

No lymph node metastasis was found by lymph node sampling in 38 AIS and 115 MIA. Many other authors reported the same situation, such as Russell [15], Wang-Yu Zhu [16], Naoki Yanagawa [4] and Kyuichi Kadota [17]. There is no such absolute standard for lymph node dissection of AIS/MIA. Ye B. also believed patients of pure ground-grass opacity (GGO) generally have no lymph node metastasis. Tumor diameter >1 cm, imaging findings with the mixed GGO or solid nodules, CEA >5 μg/L, PET-CT SUVmax >5 are predictive factors of lymph node metastasis [18]. Ye B. emphasized in another study to reduce the clinical intervention for AIS and MIA, such as segmentectomy or lymph node sampling [19]. Long Jiang also mentioned the recurrence and lymph node metastasis was rare in AIS/MIA [20]. So less clinical intervention with sublobar resection was suggested and no lymph node sampling would be warranted for both AIS and MIA, especially for those with tumor size less than 1 cm, normal serum CEA level and PET-CT SUVmax less than 5.

The follow-up chest CT scans before surgery of 30 AIS and 51 MIA patients were analyzed. 16.7% (5 in 30) AIS and 27.5% (14 in 51) MIA had mild progression in the chest CT scans. There was significant association between symptoms and more mild progression of image in chest CT. These patients with mild progression in the chest CT showed no difference of outcome with others who were stable during the follow-up before surgery. So patients who are suspicious of AIS/MIA should be given a longer follow-up time before surgery. Many patients got the surgery therapy kind of “too early”. More researches should be carried out to help copy with the over-aggressive surgery, so that doctors can give patients a general follow-up plan before surgery, taking symptoms into consideration, in the future.

Therese Phillips [21] and Jaemoon Koh [22] both found that the expression of PD-L1 was significantly associated with the tumor cell differentiation and lymph node metastasis. Ching-Yao Yang [23] also demonstrated that PD-L1 expression was significantly associated with higher grade differentiation and vascular invasion. Tomoyuki Igarashi [24] revealed the expression rate of PD-L1was higher in stage II/III invasive lung adenocarcinoma than stage I. Yixing Mao [25] got the same conclusion that PD-L1 expression was associated with lymph node metastasis and TNM stage. Yang Zhang [26] reported 1 case of AIS and 6 MIA with the all negative result of PD-L1 expression. In our study, all MIA cases were PD-L1 negative, and only one case of AIS was weak positive and there was no lymph nodes metastasis in all cases. It's maybe consistent to the early stage of cancer with less invasion and resistance to immune system. On the other hand, Shafei Wu [27] found PD-L1 was highly expressed in male patients and smokers with lung adenocarcinoma. In AIS and MIA cases, there were much more female patients and non-smokers, which could also contribute to the low expression of PD-L1 in AIS and MIA patients. But beyond that, there were few studies focus on the expression of PD-L1 in AIS or MIA, thus our study was of great meaning.

The PD-L1 expression was also widely studied in other kinds of cancers, some of which had almost the same features of PD-L1 expression as lung adenocarcinoma. For an instance, the PD-L1 expression was significant associated with higher tumor grade and lymph node invasion [28], which was consistent with the situation in lung adenocarcinoma. Another study about PD-L1 expression in breast cancer revealed the significant association between PD-L1 expression and lower histologic stage, absence of necrosis [29].

There may be some biases in our study. First, to make sure there was enough tissue, AIS/MIA cases whose tumor size larger than 1 cm were enrolled to do PD-L1 IHC analysis, resulting in selection bias. Second, women seemed to pay more attention on health or much easier to get worried about pulmonary nodules, which leaded to Berkson bias. Third, the accuracy of the diagnosis of AIS/MIA was questionable. Jennifer M. Boland has mentioned such situation where two pathologists didn't agree with each other in some diagnosis of AIS/MIA [30].

## CONCLUSIONS

There is no significance between AIS and MIA, which is remained some doubts about the meaning to distinguish AIS and MIA. In patients with AIS or MIA, female patients are much more than male, especially female without smoking. AIS and MIA almost do not express PD-L1 protein and without any lymph node metastasis. The surgery intervention should be reduced to as small as possible.

## MATERIALS AND METHODS

### Patients

We retrospectively screened 274 patients who underwent consecutive pulmonary resection surgery and were diagnosed as AIS or MIA at the SRRS hospital between 2014 to June 2016. In these patients, 77 patients were diagnosed as AIS while 197 patients as MIA according to IASLC/ATS/ERS 2011 classification [2]. The follow-up time was until 14th November, 2016. The follow-up before surgery was defined as the period between the date of first chest CT to find nodules and the date of surgery; the follow-up after surgery meant from the date of surgery to the date of latest chest CT.

### Clinical characteristics

The clinical characteristics of the patients were recorded, including age, sex, symptoms, smoking history, related past history and family history, surgical methods, nodule location, single or multiple nodules, tumor size, ALK, ROS-1, EGFR mutation and serum CEA level. Symptoms included coughing, expectoration, chest tightness, chest pain, blood-stained sputum and so on. Smoking history meant smoking in the past or present, no matter how long or how much and regardless of quit. Related past history and family history was defined as history of respiratory disease, tumors or immediate relatives with malignant tumors. Multiple nodules stand for more than one nodule found in the resection specimens. EGFR mutation, ALK, ROS-1, serum CEA level were all detected in the SRRS Hospital. The normal range of serum CEA was 0–5 μg/L.

### Immunohistochemical (IHC) analysis of PD-L1 protein

Considering IHC analysis needs adequate tumor tissue, we enrolled 17 AIS patients and 37 MIA patients to undergo IHC analysis of PD-L1 protein, whose tumor size was over 1 cm. We used 4-μm-thick sections of archival formalin-fixated, paraffin-embedded tissues. Briefly, sections were de-paraffinized using xylene followed by ethanol and then washed with distilled water. Then we incubated the slides in 0.3% hydrogen peroxide methanol solution for 10 minutes at room temperature. Each slide was heat-treated using boiled EDTA buffer retrieval solution for 20 minutes at 95–100°C, then cooled to indoor temperature for 10 minutes and put into phosphate-buffered saline (PBS PH7.4) for decolorization. Next each slide was incubated with the primary PD-L1 antibody clone E1L3N (#13684, Cell Signaling Technology, Danvers, MA) for 30 minutes. After adding enhancement solution, we incubated each slide with the secondary antibody (PV-9000, ZSGB bio company of Beijing, China) for 30 minutes. The sections were then rinsed in PBS, stained with DAB.

All IHC analysis was independently evaluated by two experienced observers (Z. T. and F. G.) who were unaware of the patients’ conditions. Cases in which the pathologists disagreed with each other, the staining category were reviewed jointly and a single consensus category was established. Tumors with staining in over or equal to 1% of tumor cells were considered as positive for PD-L1 expression, according to the large-scale clinical trial KEYNOTE-010 [9].

### Statistical analyses

The relationship of clinical features between AIS and MIA was analyzed using χ2 test. Use univariate analysis to reveal the relationship between factors like age, sex, symptoms, past history, smoking with mild progression of nodules in the chest CT before surgery. Differences were considered statistically significant at a two-sided *P*-value less than 0.05. All statistical analyses were performed using SPSS 22.

## SUPPLEMENTARY MATERIALS TABLE





## References

[1] Siegel RL, Miller KD, Jemal A (2016). Cancer statistics, 2016. CA Cancer J Clin.

[2] Travis WD, Brambilla E, Noguchi M, Nicholson AG, Geisinger KR, Yatabe Y, Beer DG, Powell CA, Riely GJ, Van Schil PE, Garg K, Austin JH, Asamura H (2011). International association for the study of lung cancer/american thoracic society/european respiratory society international multidisciplinary classification of lung adenocarcinoma. J Thorac Oncol.

[3] Aberle DR, Adams AM, Berg CD, Black WC, Clapp JD, Fagerstrom RM, Gareen IF, Gatsonis C, Marcus PM, Sicks JD (2011). Reduced lung-cancer mortality with low-dose computed tomographic screening. N Engl J Med.

[4] Yanagawa N, Shiono S, Abiko M, Ogata SY, Sato T, Tamura G (2014). The correlation of the International Association for the Study of Lung Cancer (IASLC)/American Thoracic Society (ATS)/European Respiratory Society (ERS) classification with prognosis, EGFR mutation in lung adenocarcinoma. Ann Thorac Surg.

[5] Sato S, Motoi N, Hiramatsu M, Miyauchi E, Ono H, Saito Y, Nagano H, Ninomiya H, Inamura K, Uehara H, Mun M, Sakao Y, Okumura S (2015). Pulmonary adenocarcinoma in situ: analyses of a large series with reference to smoking, driver mutations, and receptor tyrosine kinase pathway activation. Am J Surg Pathol.

[6] Okazaki T, Honjo T (2007). PD-1, PD-1 ligands: from discovery to clinical application. Int Immunol.

[7] Borghaei H, Paz-Ares L, Horn L, Spigel DR, Steins M, Ready NE, Chow LQ, Vokes EE, Felip E, Holgado E, Barlesi F, Kohlhaufl M, Arrieta O (2015). Nivolumab versus Docetaxel in Advanced Nonsquamous Non-Small-Cell Lung Cancer. N Engl J Med.

[8] Brahmer J, Reckamp KL, Baas P, Crino L, Eberhardt WE, Poddubskaya E, Antonia S, Pluzanski A, Vokes EE, Holgado E, Waterhouse D, Ready N, Gainor J (2015). Nivolumab versus Docetaxel in Advanced Squamous-Cell Non-Small-Cell Lung Cancer. N Engl J Med.

[9] Reck M, Rodriguez-Abreu D, Robinson AG, Hui R, Csoszi T, Fulop A, Gottfried M, Peled N, Tafreshi A, Cuffe S, O’Brien M, Rao S, Hotta K (2016). Pembrolizumab versus Chemotherapy for PD-L1-Positive Non-Small-Cell Lung Cancer. N Engl J Med.

[10] Herbst RS, Baas P, Kim DW, Felip E, Perez-Gracia JL, Han JY, Molina J, Kim JH, Arvis CD, Ahn MJ, Majem M, Fidler MJ, de Castro GJ (2016). Pembrolizumab versus docetaxel for previously treated, PD-L1-positive, advanced non-small-cell lung cancer (KEYNOTE-010): a randomised controlled trial. Lancet.

[11] Brahmer JR, Tykodi SS, Chow LQ, Hwu WJ, Topalian SL, Hwu P, Drake CG, Camacho LH, Kauh J, Odunsi K, Pitot HC, Hamid O, Bhatia S (2012). Safety and activity of anti-PD-L1 antibody in patients with advanced cancer. N Engl J Med.

[12] Topalian SL, Hodi FS, Brahmer JR, Gettinger SN, Smith DC, McDermott DF, Powderly JD, Carvajal RD, Sosman JA, Atkins MB, Leming PD, Spigel DR, Antonia SJ (2012). Safety, activity, and immune correlates of anti-PD-1 antibody in cancer. N Engl J Med.

[13] Behera M, Owonikoko TK, Gal AA, Steuer CE, Kim S, Pillai RN, Khuri FR, Ramalingam SS, Sica GL (2016). Lung Adenocarcinoma Staging Using the 2011 IASLC/ATS/ERS Classification: A Pooled Analysis of Adenocarcinoma In Situ, Minimally Invasive Adenocarcinoma. Clin Lung Cancer.

[14] Bracci PM, Sison J, Hansen H, Walsh KM, Quesenberry CP, Raz DJ, Wrensch M, Wiencke JK (2012). Cigarette smoking associated with lung adenocarcinoma in situ in a large case-control study (SFBALCS). J Thorac Oncol.

[15] Russell PA, Wainer Z, Wright GM, Daniels M, Conron M, Williams RA (2011). Does lung adenocarcinoma subtype predict patient survival?: A clinicopathologic study based on the new International Association for the Study of Lung Cancer/American Thoracic Society/European Respiratory Society international multidisciplinary lung adenocarcinoma classification. J Thorac Oncol.

[16] Zhu WY, Tan LL, Wang ZY, Wang SJ, Xu LY, Yu W, Chen ZJ, Zhang YK (2016). Clinical characteristics and advantages of primary peripheral micro-sized lung adenocarcinoma over small-sized lung adenocarcinoma. Eur J Cardiothorac Surg.

[17] Kadota K, Villena-Vargas J, Yoshizawa A, Motoi N, Sima CS, Riely GJ, Rusch VW, Adusumilli PS, Travis WD (2014). Prognostic significance of adenocarcinoma in situ, minimally invasive adenocarcinoma, and nonmucinous lepidic predominant invasive adenocarcinoma of the lung in patients with stage I disease. Am J Surg Pathol.

[18] Ye B, Feng J, Pan XF, Yang Y, Geng JF, Cao KJ, Zhao H, Hu DZ (2013). [Correlation analysis between imaging features and lymph node metastasis in T1a lung adenocarcinoma]. [Article in Chinese]. Zhonghua Wai Ke Za Zhi.

[19] Ye B, Cheng M, Li W, Ge XX, Geng JF, Feng J, Yang Y, Hu DZ (2014). Predictive factors for lymph node metastasis in clinical stage IA lung adenocarcinoma. Ann Thorac Surg.

[20] Jiang L, Yin W, Peng G, Wang W, Zhang J, Liu Y, Zhong S, He Q, Liang W, He J (2015). Prognosis and status of lymph node involvement in patients with adenocarcinoma in situ and minimally invasive adenocarcinoma-a systematic literature review and pooled-data analysis. J Thorac Dis.

[21] Phillips T, Simmons P, Inzunza HD, Cogswell J, Novotny JJ, Taylor C, Zhang X (2015). Development of an automated PD-L1 immunohistochemistry (IHC) assay for non-small cell lung cancer. Appl Immunohistochem Mol Morphol.

[22] Koh J, Go H, Keam B, Kim MY, Nam SJ, Kim TM, Lee SH, Min HS, Kim YT, Kim DW, Jeon YK, Chung DH (2015). Clinicopathologic analysis of programmed cell death-1 and programmed cell death-ligand 1 and 2 expressions in pulmonary adenocarcinoma: comparison with histology and driver oncogenic alteration status. Mod Pathol.

[23] Yang CY, Lin MW, Chang YL, Wu CT, Yang PC (2014). Programmed cell death-ligand 1 expression in surgically resected stage I pulmonary adenocarcinoma and its correlation with driver mutations and clinical outcomes. Eur J Cancer.

[24] Igarashi T, Teramoto K, Ishida M, Hanaoka J, Daigo Y (2016). Scoring of PD-L1 expression intensity on pulmonary adenocarcinomas and the correlations with clinicopathological factors. ESMO Open.

[25] Mao Y, Li W, Chen K, Xie Y, Liu Q, Yao M, Duan W, Zhou X, Liang R, Tao M (2015). B7-H1 and B7-H3 are independent predictors of poor prognosis in patients with non-small cell lung cancer. Oncotarget.

[26] Zhang Y, Wang L, Li Y, Pan Y, Wang R, Hu H, Li H, Luo X, Ye T, Sun Y, Chen H (2014). Protein expression of programmed death 1 ligand 1 and ligand 2 independently predict poor prognosis in surgically resected lung adenocarcinoma. Onco Targets Ther.

[27] Wu S, Shi X, Sun J, Liu Y, Luo Y, Liang Z, Wang J, Zeng X (2017). The significance of programmed cell death ligand 1 expression in resected lung adenocarcinoma. Oncotarget.

[28] Muenst S, Schaerli AR, Gao F, Daster S, Trella E, Droeser RA, Muraro MG, Zajac P, Zanetti R, Gillanders WE, Weber WP, Soysal SD (2014). Expression of programmed death ligand 1 (PD-L1) is associated with poor prognosis in human breast cancer. Breast Cancer Res Treat.

[29] Tsang JY, Au WL, Lo KY, Ni YB, Hlaing T, Hu J, Chan SK, Chan KF, Cheung SY, Tse GM (2017). PD-L1 expression and tumor infiltrating PD-1+ lymphocytes associated with outcome in HER2+ breast cancer patients. Breast Cancer Res Treat.

[30] Boland JM, Froemming AT, Wampfler JA, Maldonado F, Peikert T, Hyland C, de Andrade M, Aubry MC, Yang P, Yi ES (2016). Adenocarcinoma in situ, minimally invasive adenocarcinoma, and invasive pulmonary adenocarcinoma--analysis of interobserver agreement, survival, radiographic characteristics, and gross pathology in 296 nodules. Hum Pathol.

