# Supplementary material for: Clinical characteristics and programmed cell death ligand-1 expression in adenocarcinoma in situ and minimally invasive adenocarcinoma of lung
Source: Oncotarget. 2017 Oct 26;8(58):97801–10. doi: 10.18632/oncotarget.22082 (PMC5716692; doi:10.18632/oncotarget.22082)
Supplement: Supplementary file 1 [file oncotarget-08-97801-s001.pdf]

Clinical characteristics and programmed cell death ligand-1 expression in adenocarcinoma *in situ* and minimally invasive adenocarcinoma of lung

SUPPLEMENTARY MATERIALS

Supplementary Table 1: The detailed information of AIS/MIA patients. See Supplementary_Table_1
