# Supplementary material for: Clinical characteristics and programmed cell death ligand-1 expression in adenocarcinoma in situ and minimally invasive adenocarcinoma of lung
Source: Oncotarget. 2017 Oct 26;8(58):97801–10. doi: 10.18632/oncotarget.22082 (PMC5716692; doi:10.18632/oncotarget.22082)
Supplement: Supplementary file 2 [file oncotarget-08-97801-s002.docx]

| number | **Patient ID** | **sex** | **age** | **time** | **location** | **diagnosis** |
| --- | --- | --- | --- | --- | --- | --- |
| 1 | 3634339 | M | 48 | 2016/1/5 | RML | AIS |
| 2 | 3770630 | F | 53 | 2016/5/11 | LUL | AIS |
| 3 | 2064830 | F | 47 | 2016/3/9 | RLL | AIS |
| 4 | 3114660 | M | 64 | 2016/3/23 | RUL | AIS |
| 5 | 3712366 | F | 52 | 2016/4/18 | RUL | AIS |
| 6 | 680492 | F | 46 | 2016/1/20 | LLL | AIS |
| 7 | 2904507 | F | 80 | 2015/5/4 | LLL, LLL | AIS |
| 8 | 3343128 | F | 58 | 2015/4/9 | RUL | AIS |
| 9 | 3319733 | F | 44 | 2015/4/23 | RLL | AIS |
| 10 | 3376538 | M | 39 | 2015/3/3 | RML | AIS |
| 11 | 2460448 | F | 43 | 2015/3/25 | LUL | AIS |
| 12 | 3624049 | F | 47 | 2015/12/15 | RML | AIS |
| 13 | 3401461 | F | 65 | 2015/3/24 | LLL, LUL, LLL | AIS |
| 14 | 3365867 | F | 41 | 2015/3/18 | LLL | AIS |
| 15 | 3061874 | F | 48 | 2015/4/9 | RUL | AIS |
| 16 | 2557694 | F | 74 | 2015/5/27 | LUL | AIS |
| 17 | 3361199 | F | 43 | 2015/2/3 | RLL | AIS |
| 18 | 3345103 | F | 70 | 2015/4/21 | RML | AIS |
| 19 | 2797612 | F | 35 | 2015/2/25 | RLL | AIS |
| 20 | 3598915 | F | 41 | 2015/11/18 | RUL | AIS |
| 21 | 1058828 | F | 47 | 2015/6/30 | LLL | AIS |
| 22 | 8143930 | F | 52 | 2015/11/4 | LUL | AIS |
| 23 | 2903139 | F | 51 | 2015/1/6 | RUL | AIS |
| 24 | 3197443 | F | 41 | 2015/6/15 | LLL | AIS |
| 25 | 1672049 | F | 56 | 2015/11/23 | LUL | AIS |
| 26 | 3059774 | M | 37 | 2015/9/15 | LUL | AIS |
| 27 | 3156174 | F | 56 | 2015/4/2 | RUL | AIS |
| 28 | 3616772 | F | 53 | 2015/12/8 | LUL | AIS |
| 29 | 3425087 | M | 45 | 2015/4/23 | LUL | AIS |
| 30 | 3589885 | F | 49 | 2015/10/27 | LUL | AIS |
| 31 | 206078 | F | 48 | 2015/3/30 | RUL | AIS |
| 32 | 1958777 | M | 52 | 2015/6/19 | RML | AIS |
| 33 | 3337177 | F | 50 | 2015/6/22 | LUL | AIS |
| 34 | 2760713 | M | 65 | 2014/10/31 | LUL | AIS |
| 35 | 3149105 | M | 67 | 2014/6/24 | RUL | AIS |
| 36 | 1825506 | M | 48 | 2014/6/18 | RUL | AIS |
| 37 | 2812307 | F | 41 | 2014/6/25 | LUL | AIS |
| 38 | 1330512 | F | 53 | 2014/4/1 | RLL | AIS |
| 39 | 3208512 | F | 53 | 2014/7/22 | LUL | AIS |
| 40 | 3254215 | M | 57 | 2014/9/2 | LUL | AIS |
| 41 | 3237616 | F | 60 | 2014/8/12 | LUL | AIS |
| 42 | 1977816 | F | 57 | 2014/7/3 | RUL, RLL | AIS |
| 43 | 2478417 | F | 66 | 2014/4/22 | LUL | AIS |
| 44 | 3107320 | F | 36 | 2014/3/20 | RLL | AIS |
| 45 | 1667026 | M | 57 | 2014/9/1 | RUL | AIS |
| 46 | 1329126 | F | 46 | 2014/7/23 | RUL, RLL | AIS |
| 47 | 1333030 | M | 63 | 2014/6/25 | RUL | AIS |
| 48 | 3003732 | M | 61 | 2014/10/15 | RUL | AIS |
| 49 | 3182334 | M | 67 | 2014/6/26 | RUL | AIS |
| 50 | 1384336 | F | 56 | 2014/11/11 | RUL | AIS |
| 51 | 2078738 | M | 58 | 2014/6/9 | LUL | AIS |
| 52 | 3205749 | M | 53 | 2014/7/24 | LUL | AIS |
| 53 | 2610150 | F | 55 | 2014/5/2 | RML | AIS |
| 54 | 3010752 | M | 62 | 2014/7/24 | RML | AIS |
| 55 | 3190452 | F | 61 | 2014/6/30 | RLL | AIS |
| 56 | 1784952 | M | 62 | 2014/9/10 | RLL | AIS |
| 57 | 659552 | F | 65 | 2014/5/22 | RLL, RUL | AIS |
| 58 | 2336356 | F | 51 | 2014/11/5 | RUL, RUL | AIS |
| 59 | 2431762 | M | 63 | 2014/12/8 | RUL | AIS |
| 60 | 1103067 | M | 66 | 2014/3/20 | LUL,LUL | AIS |
| 61 | 2715067 | F | 25 | 2014/2/19 | LUL | AIS |
| 62 | 3138467 | F | 30 | 2014/5/15 | LUL | AIS |
| 63 | 2840069 | F | 60 | 2014/11/11 | RUL | AIS |
| 64 | 3130674 | F | 47 | 2014/4/23 | LLL | AIS |
| 65 | 8046776 | F | 48 | 2014/8/28 | LLL | AIS |
| 66 | 243280 | M | 38 | 2014/7/29 | RUL | AIS |
| 67 | 2853181 | F | 39 | 2014/1/13 | RUL | AIS |
| 68 | 1999883 | F | 59 | 2014/3/12 | LLL | AIS |
| 69 | 8050786 | F | 35 | 2014/9/3 | RUL | AIS |
| 70 | 210286 | F | 63 | 2014/5/13 | LUL | AIS |
| 71 | 2701087 | F | 46 | 2014/8/27 | LUL | AIS |
| 72 | 2734987 | F | 36 | 2014/7/16 | LUL | AIS |
| 73 | 3297287 | F | 51 | 2014/10/24 | RML | AIS |
| 74 | 3252789 | M | 70 | 2014/9/3 | RLL | AIS |
| 75 | 1634493 | F | 47 | 2014/7/8 | RUL | AIS |
| 76 | 3214799 | F | 39 | 2014/7/31 | LLL, LLL | AIS |
| 77 | 2335750 | F | 43 | 2014/4/2 | RUL, LUL, LLL, LLL | AIS |
|  |  |  |  |  |  |  |
| number | Patient ID | sex | age | time | location | diagnosis |
| 1 | 1355715 | F | 46 | 2016/5/18 | RLL, RML | MIA |
| 2 | 3668929 | F | 41 | 2016/2/25 | LLL, LUL, LUL | MIA |
| 3 | 3636148 | F | 67 | 2016/1/7 | LUL, LUL, LLL | MIA |
| 4 | 3668555 | F | 37 | 2016/1/29 | LLL | MIA |
| 5 | 3583572 | F | 46 | 2016/1/26 | RUL | MIA |
| 6 | 3639573 | M | 55 | 2016/1/26 | RUL | MIA |
| 7 | 2290481 | M | 63 | 2016/1/20 | RUL | MIA |
| 8 | 2880600 | F | 34 | 2016/5/12 | RLL, RLL, RLL | MIA |
| 9 | 3484602 | M | 56 | 2016/3/3 | RUL | MIA |
| 10 | 1003904 | M | 39 | 2016/3/2 | RLL | MIA |
| 11 | 3383105 | F | 52 | 2016/6/10 | RML | MIA |
| 12 | 3688005 | M | 60 | 2016/3/1 | RLL | MIA |
| 13 | 3783707 | F | 44 | 2016/6/1 | RML | MIA |
| 14 | 3713308 | M | 53 | 2016/3/22 | RUL | MIA |
| 15 | 3662809 | F | 45 | 2016/6/8 | LLL | MIA |
| 16 | 3818710 | F | 55 | 2016/6/16 | RUL, RLL | MIA |
| 17 | 3613612 | F | 41 | 2016/4/17 | LUL | MIA |
| 18 | 3723512 | F | 33 | 2016/3/29 | LUL | MIA |
| 19 | 2630414 | F | 58 | 2016/3/29 | LUL, LLL | MIA |
| 20 | 2744414 | F | 55 | 2016/2/24 | RUL | MIA |
| 21 | 3325019 | F | 29 | 2015/12/25 | LLL | MIA |
| 22 | 3454320 | F | 51 | 2016/5/4 | RUL | MIA |
| 23 | 2713322 | F | 38 | 2015/12/30 | RUL | MIA |
| 24 | 1477323 | M | 61 | 2015/12/30 | RUL, RLL | MIA |
| 25 | 1498924 | M | 46 | 2016/2/17 | RLL | MIA |
| 26 | 3763527 | F | 40 | 2016/5/6 | RUL | MIA |
| 27 | 3745228 | F | 48 | 2016/5/3 | LLL | MIA |
| 28 | 142529 | M | 46 | 2016/3/9 | LUL, LLL | MIA |
| 29 | 1092132 | F | 56 | 2016/1/14 | LLL | MIA |
| 30 | 3662334 | F | 47 | 2016/5/27 | RLL | MIA |
| 31 | 3709240 | M | 55 | 2016/3/22 | LUL | MIA |
| 32 | 3749343 | F | 48 | 2016/4/21 | RML | MIA |
| 33 | 3378545 | M | 70 | 2016/2/24 | RUL, RML | MIA |
| 34 | 3722152 | F | 53 | 2016/4/13 | RLL | MIA |
| 35 | 1244152 | M | 46 | 2016/3/15 | RUL | MIA |
| 36 | 3644352 | F | 51 | 2016/1/7 | LUL | MIA |
| 37 | 3292856 | F | 54 | 2016/3/24 | LUL, LUL | MIA |
| 38 | 3730659 | F | 54 | 2016/4/14 | RUL, RUL | MIA |
| 39 | 409959 | F | 44 | 2016/4/27 | LUL, LUL | MIA |
| 40 | 3758560 | F | 45 | 2016/5/5 | RUL | MIA |
| 41 | 3819860 | F | 36 | 2016/6/21 | LLL | MIA |
| 42 | 3714161 | F | 64 | 2016/4/5 | LUL | MIA |
| 43 | 3786661 | F | 60 | 2016/5/24 | RUL, RML | MIA |
| 44 | 3474162 | F | 54 | 2016/6/22 | LUL | MIA |
| 45 | 3656863 | M | 73 | 2016/4/6 | RUL | MIA |
| 46 | 3681865 | F | 65 | 2016/3/18 | RUL | MIA |
| 47 | 3639366 | F | 30 | 2016/1/15 | RML | MIA |
| 48 | 3706470 | F | 45 | 2016/3/30 | LUL | MIA |
| 49 | 3656275 | F | 46 | 2016/2/19 | RUL | MIA |
| 50 | 3679078 | M | 47 | 2016/2/17 | RLL | MIA |
| 51 | 2003880 | M | 63 | 2016/3/22 | LUL | MIA |
| 52 | 3799080 | F | 43 | 2016/6/8 | LUL | MIA |
| 53 | 3678785 | F | 53 | 2016/2/18 | LUL | MIA |
| 54 | 3770986 | M | 55 | 2016/6/15 | RLL | MIA |
| 55 | 3732288 | F | 53 | 2016/6/7 | RUL | MIA |
| 56 | 1129589 | F | 52 | 2016/2/18 | LLL | MIA |
| 57 | 3573191 | F | 44 | 2016/4/14 | RUL | MIA |
| 58 | 3716991 | F | 46 | 2016/5/10 | RUL | MIA |
| 59 | 3787794 | M | 43 | 2016/5/25 | RLL | MIA |
| 60 | 3706698 | F | 35 | 2016/6/21 | RLL | MIA |
| 61 | 3430210 | F | 42 | 2015/6/18 | LUL | MIA |
| 62 | 3487010 | F | 41 | 2015/6/25 | RUL | MIA |
| 63 | 3599618 | M | 51 | 2015/11/10 | RUL | MIA |
| 64 | 3346224 | F | 66 | 2014/12/30 | RLL | MIA |
| 65 | 44339 | F | 63 | 2015/3/12 | RLL | MIA |
| 66 | 3197041 | M | 64 | 2015/3/11 | RLL | MIA |
| 67 | 3230243 | F | 79 | 2015/5/4 | LUL | MIA |
| 68 | 3332343 | F | 56 | 2015/1/5 | RUL, RUL | MIA |
| 69 | 3410244 | M | 52 | 2015/3/31 | RUL | MIA |
| 70 | 1571746 | F | 56 | 2015/2/10 | LUL | MIA |
| 71 | 3383149 | M | 52 | 2015/2/26 | RUL | MIA |
| 72 | 3360957 | M | 65 | 2015/1/21 | RML | MIA |
| 73 | 3387557 | F | 63 | 2015/3/5 | RUL | MIA |
| 74 | 3434965 | M | 61 | 2015/5/12 | RUL | MIA |
| 75 | 2368768 | F | 76 | 2015/4/24 | LUL | MIA |
| 76 | 3347871 | F | 51 | 2014/12/31 | RUL | MIA |
| 77 | 636376 | M | 59 | 2015/1/28 | RUL | MIA |
| 78 | 3391978 | F | 40 | 2015/3/9 | RUL | MIA |
| 79 | 2666078 | F | 39 | 2015/3/18 | RLL | MIA |
| 80 | 3386779 | F | 46 | 2015/3/5 | RUL, RLL | MIA |
| 81 | 3412193 | F | 57 | 2015/3/31 | LLL | MIA |
| 82 | 3315499 | M | 40 | 2015/3/20 | RUL | MIA |
| 83 | 3352700 | F | 67 | 2015/1/13 | RML | MIA |
| 84 | 1432900 | M | 78 | 2015/12/15 | RLL | MIA |
| 85 | 1987800 | F | 59 | 2015/11/25 | RML | MIA |
| 86 | 3491504 | F | 52 | 2015/7/7 | RUL | MIA |
| 87 | 3585608 | F | 46 | 2015/10/20 | RLL | MIA |
| 88 | 3453611 | M | 46 | 2015/6/11 | RLL | MIA |
| 89 | 3556111 | F | 53 | 2015/9/14 | LUL | MIA |
| 90 | 8016511 | F | 56 | 2015/5/8 | LUL | MIA |
| 91 | 1670512 | F | 56 | 2015/12/11 | RUL | MIA |
| 92 | 230514 | F | 55 | 2015/8/7 | RUL | MIA |
| 93 | 2791814 | F | 70 | 2015/4/15 | RUL | MIA |
| 94 | 3601715 | M | 71 | 2015/11/26 | LUL | MIA |
| 95 | 3494015 | M | 57 | 2015/7/16 | RUL | MIA |
| 96 | 3359815 | F | 47 | 2015/4/9 | RUL | MIA |
| 97 | 3610116 | M | 37 | 2015/11/30 | LLL | MIA |
| 98 | 3315616 | F | 37 | 2015/3/18 | LLL | MIA |
| 99 | 3115618 | F | 44 | 2015/7/30 | LUL | MIA |
| 100 | 3568918 | F | 29 | 2015/10/8 | LUL, LUL | MIA |
| 101 | 2739420 | F | 46 | 2015/4/8 | LUL | MIA |
| 102 | 3474424 | F | 61 | 2015/6/11 | RUL | MIA |
| 103 | 3483725 | F | 42 | 2015/6/25 | RUL, RLL | MIA |
| 104 | 677130 | F | 48 | 2015/11/20 | RUL | MIA |
| 105 | 3469830 | F | 59 | 2015/6/10 | RUL, RML | MIA |
| 106 | 3454531 | F | 71 | 2015/6/29 | RUL | MIA |
| 107 | 3504431 | F | 58 | 2015/7/23 | RUL | MIA |
| 108 | 3428731 | F | 33 | 2015/4/28 | LLL | MIA |
| 109 | 3470232 | F | 59 | 2015/6/9 | RUL | MIA |
| 110 | 3411932 | M | 52 | 2015/4/8 | LLL | MIA |
| 111 | 3609534 | F | 31 | 2015/11/30 | RML | MIA |
| 112 | 3595536 | M | 44 | 2015/12/9 | LUL | MIA |
| 113 | 3456336 | M | 53 | 2015/5/21 | LLL | MIA |
| 114 | 3314442 | F | 82 | 2014/12/2 | RLL | MIA |
| 115 | 3498543 | F | 54 | 2015/7/14 | RUL, RLL | MIA |
| 116 | 3504444 | F | 51 | 2015/12/15 | LLL | MIA |
| 117 | 2564547 | F | 44 | 2015/10/29 | RUL | MIA |
| 118 | 2777047 | M | 69 | 2015/8/6 | RLL | MIA |
| 119 | 3433648 | F | 56 | 2015/5/7 | RLL | MIA |
| 120 | 3494648 | F | 53 | 2015/7/7 | LUL | MIA |
| 121 | 3477448 | M | 48 | 2015/6/17 | LUL | MIA |
| 122 | 3560549 | M | 65 | 2015/9/18 | RUL | MIA |
| 123 | 2341149 | F | 35 | 2015/12/1 | RUL | MIA |
| 124 | 528650 | M | 69 | 2015/6/23 | LLL | MIA |
| 125 | 3339350 | F | 51 | 2015/8/21 | RUL | MIA |
| 126 | 3521852 | F | 46 | 2015/8/4 | RUL,RLL,RML,RUL | MIA |
| 127 | 1800154 | F | 49 | 2015/7/14 | LUL | MIA |
| 128 | 3392556 | F | 59 | 2015/6/1 | LLL, LLL | MIA |
| 129 | 1093956 | F | 59 | 2015/7/7 | RUL | MIA |
| 130 | 8142658 | F | 43 | 2015/10/15 | LUL, LLL | MIA |
| 131 | 3272458 | F | 61 | 2015/8/26 | RUL | MIA |
| 132 | 3020963 | F | 44 | 2015/12/28 | LLL | MIA |
| 133 | 3486363 | F | 44 | 2015/7/3 | RLL | MIA |
| 134 | 3563364 | F | 54 | 2015/9/23 | RUL | MIA |
| 135 | 1074164 | F | 63 | 2015/8/17 | RUL | MIA |
| 136 | 3203774 | F | 73 | 2015/3/24 | RML | MIA |
| 137 | 3590174 | F | 47 | 2015/11/20 | LUL | MIA |
| 138 | 760673 | M | 53 | 2015/10/28 | RUL, RUL | MIA |
| 139 | 3472672 | F | 43 | 2015/6/18 | LUL | MIA |
| 140 | 3595170 | F | 52 | 2015/11/6 | RUL | MIA |
| 141 | 1306495 | F | 35 | 2015/8/12 | RUL | MIA |
| 142 | 3366893 | M | 59 | 2015/1/28 | RUL | MIA |
| 143 | 2069592 | M | 52 | 2015/7/13 | LUL | MIA |
| 144 | 2710092 | F | 70 | 2015/10/8 | RUL | MIA |
| 145 | 3479788 | F | 47 | 2015/7/2 | LUL | MIA |
| 146 | 3519588 | F | 47 | 2015/9/2 | RUL | MIA |
| 147 | 3054788 | F | 56 | 2015/11/17 | LLL | MIA |
| 148 | 3614184 | F | 52 | 2015/12/8 | RLL | MIA |
| 149 | 1177281 | F | 58 | 2015/1/13 | RUL | MIA |
| 150 | 3489080 | F | 62 | 2015/7/1 | LUL | MIA |
| 151 | 3285380 | F | 50 | 2015/3/3 | LUL | MIA |
| 152 | 1802580 | F | 54 | 2015/11/24 | RML | MIA |
| 153 | 3584779 | F | 46 | 2015/11/3 | RML, RLL | MIA |
| 154 | 3593179 | F | 59 | 2015/12/3 | RUL | MIA |
| 155 | 3024876 | M | 71 | 2015/6/9 | LUL | MIA |
| 156 | 2003865 | M | 43 | 2015/10/6 | RLL | MIA |
| 157 | 2571700 | F | 53 | 2014/11/25 | LUL | MIA |
| 158 | 2323801 | F | 36 | 2014/11/4 | RUL | MIA |
| 159 | 3300524 | F | 76 | 2014/10/29 | LUL | MIA |
| 160 | 3143903 | M | 52 | 2014/5/14 | LUL | MIA |
| 161 | 2747605 | F | 58 | 2014/12/11 | RML | MIA |
| 162 | 8054916 | M | 40 | 2014/12/26 | RUL | MIA |
| 163 | 3291518 | F | 40 | 2014/10/22 | RLL | MIA |
| 164 | 3163020 | F | 66 | 2014/5/26 | LUL | MIA |
| 165 | 1439721 | M | 65 | 2014/7/28 | LUL | MIA |
| 166 | 3167024 | F | 47 | 2014/5/28 | RUL, RUL | MIA |
| 167 | 3299324 | M | 53 | 2014/10/29 | RLL, LLL | MIA |
| 168 | 1808925 | M | 63 | 2014/11/18 | RUL, RUL, RLL | MIA |
| 169 | 1849731 | F | 56 | 2014/10/16 | LUL | MIA |
| 170 | 2861239 | F | 38 | 2014/12/5 | RUL | MIA |
| 171 | 3197148 | M | 35 | 2014/7/22 | LUL, LLL | MIA |
| 172 | 1263949 | F | 47 | 2014/5/28 | LUL, LLL | MIA |
| 173 | 990454 | M | 33 | 2014/12/9 | LUL | MIA |
| 174 | 3003668 | F | 73 | 2014/12/19 | RUL | MIA |
| 175 | 3174769 | F | 48 | 2014/6/12 | RUL | MIA |
| 176 | 1695569 | M | 47 | 2014/10/13 | RUL | MIA |
| 177 | 2354571 | M | 75 | 2014/6/17 | RLL | MIA |
| 178 | 647671 | F | 54 | 2014/12/8 | LLL | MIA |
| 179 | 3197072 | F | 60 | 2014/9/25 | RUL,RLL | MIA |
| 180 | 3294674 | F | 56 | 2014/10/24 | RLL | MIA |
| 181 | 2296975 | M | 35 | 2014/7/4 | RML | MIA |
| 182 | 1948781 | M | 27 | 2014/2/12 | RML | MIA |
| 183 | 230785 | F | 53 | 2014/3/28 | RUL | MIA |
| 184 | 2933986 | F | 45 | 2014/8/7 | RLL | MIA |
| 185 | 1421688 | F | 64 | 2014/8/19 | RLL | MIA |
| 186 | 3188094 | F | 42 | 2014/7/1 | RUL | MIA |
| 187 | 2183996 | F | 39 | 2014/5/9 | RLL | MIA |
| 188 | 3320798 | F | 43 | 2014/12/9 | RLL | MIA |
| 189 | 3136399 | M | 70 | 2014/11/10 | LLL, LLL | MIA |
| 190 | 3691218 | F | 59 | 2016/3/2 | RUL, LLL, LUL | MIA |
| 191 | 919841 | M | 62 | 2016/3/2 | RML, LUL, LLL | MIA |
| 192 | 1293554 | M | 63 | 2015/12/15 | RUL, LUL | MIA |
| 193 | 3595091 | F | 53 | 2015/11/3 | LLL, RUL | MIA |
| 194 | 8147020 | F | 47 | 2016/1/12 | RUL, RML | MIA |
| 195 | 3682501 | F | 58 | 2016/2/26 | LUL, RUL, RML | MIA |
| 196 | 3598067 | M | 54 | 2015/11/13 | RUL, RUL, LLL | MIA |
| 197 | 230491 | F | 53 | 2014/6/18 | LUL, RLL | MIA |
